# Effective Adsorption and Removal of Phosphate from Aqueous Solutions and Eutrophic Water by Fe-based MOFs of MIL-101

**DOI:** 10.1038/s41598-017-03526-x

**Published:** 2017-06-12

**Authors:** Qiying Xie, Yan Li, Zhaoling Lv, Hang Zhou, Xiangjun Yang, Jing Chen, Hong Guo

**Affiliations:** 1 School of Chemistry Science and Engineering, Yunnan University, Kunming, 650091 Yunnan China; 2 Yunnan Key Laboratory of Micro/Nano Materials & Technology, Kunming, 650091 Yunnan China

## Abstract

Although many efforts have been devoted to the adsorptive removal of phosphate from aqueous solutions and eutrophic water, it is still highly desirable to develop novel adsorbents with high adsorption capacities. In this study, Fe-based metal-organic frameworks (MOFs), MIL-101 and NH_2_-MIL-101, are fabricated through a general facile strategy. Their performance as an adsorbent for phosphate removal is investigated. Experiments are performed to study the effects of various factors on the phosphate adsorption, including adsorbent dosage, contact time and co-existing ions. Both MIL-101(Fe) and NH_2_-MIL-101(Fe) show highly effective removal of phosphates from aqueous solutions, and the concentration of phosphates decrease sharply from the initial 0.60 mg·L^−1^ to 0.045 and 0.032 mg·L^−1^, respectively, within just 30 min of exposure. The adsorption kinetics and adsorption isotherms reveal that NH_2_-MIL-101(Fe) has higher adsorption capacity than MIL-101(Fe) possibly due to the amine group. Furthermore, the Fe-based MOFs also exhibit a high selectivity towards phosphate over other anions such as chloride, bromide, nitrate and sulfate. Particularly, the prepared Fe-based MIL-101 materials are also capable of adsorbing phosphate in an actual eutrophic water sample and display better removal effect.

## Introduction

Phosphates are widely used in many industries such as food, agriculture, beverage and detergent. The excessive use of phosphorus has resulted in a large amount of pollution and environment problems, such as severe eutrophication which contributes to aquatic species death, algal bloom and parasite infections. Therefore, it has become a global necessity to efficiently decontaminate phosphates with minimal environmental impact. Many strategies have been reported for the efficient elimination of phosphates from aqueous solutions including enzymatic biodegradation^1^, adsorption^2^, electrochemistry^3^, precipitation and floatation^4^. Among these, the adsorption technique is the most widely employed method for removal of phosphates due to its environmentally safe process, simple and fast operation, and low cost^5, 6^. Developing new adsorbents with high adsorption capacities is of great significance for the effective adsorption and removal of phosphate from the environment.

In the past decade, metal-organic frameworks (MOFs) have emerged as a new class of organic-inorganic hybrid functional materials with high porosity, large surface area and morphology. The properties of these MOFs can be easily tuned by selecting different metal ions and organic bridging ligands^7–9^, and they have been developed as a new class of solid adsorbents. Compared to conventional solid adsorbents such as mesoporous silica materials^10, 11^ and activated carbon^12, 13^, MOFs exhibit more virtues such as versatile framework compositions, exposed active sites, tunable pore sizes, and large specific surface areas. MOFs have been demonstrated to be stable and show good adsorption ability for the removal of various pollutants from aqueous solutions such as heavy metal ions^14^, organic dyes^15, 16^, phenols^17^, oil droplets^18^ and humic acid^19^. However, to the best of our knowledge, few studies have been reported on the removal of phosphate from water by MOFs via adsorption, because this process not only needs adequate pore size of MOFs but also specific active sites. Recently, Gu *et al*.^20^ fabricated Zr-based MOFs of UiO-67 which showed effective adsorption and enhanced removal of organophosphorus pesticides from aqueous solutions. Lin and coworkers^21^ reported another type of Zr-based MOFs which functioned as highly selective adsorbents for the removal of phosphate from water and urine. These results prompted us to carry out the corresponding work on removal of phosphates from wastewater by MOFs. However, these previous studies are in fact mostly focused on Zr-based materials with high cost. So it is desirable to design and construct new structures with low cost such as Fe-based MOFs with exceptional stability.

Fe-based MOFs are selected in particular because they are considered to be environmentally friendly and Fe is relatively abundant in the earth’s crust. Moreover, MOFs can be easily functionalized and customized by selecting different ligands. Particularly, amine functional groups are known to exhibit enhanced phosphate adsorption^22^. Herein, in this study, the advantages of the MOFs and amine functional groups are well integrated to solve the problems in the separation and recovery of phosphates from water. Two Fe-based MOFs, MIL-101(Fe) and NH_2_-MIL-101(Fe), were synthesized with and without amine groups to examine the effect of amine group on the phosphate adsorption. The physicochemical properties of MIL-101(Fe) and NH_2_-MIL-101(Fe) were measured by X-ray diffraction (XRD), Fourier transform infrared spectroscopy (FTIR), scanning electron microscopy (SEM), and X-ray photoelectron spectroscopy (XPS). The effects of different factors on the phosphate removal efficiency and adsorption capacity, such as adsorbent dosage, contact time, initial concentration, co-existing ions and recyclability, were also studied. The adsorption kinetics and isotherm models were used for evaluating the experimental data. Moreover, a probable mechanism for the phosphate adsorption was proposed.

## Results and Discussion

### Characterization of MIL-101(Fe) and NH_2_-MIL-101(Fe)

**Figure 1:**
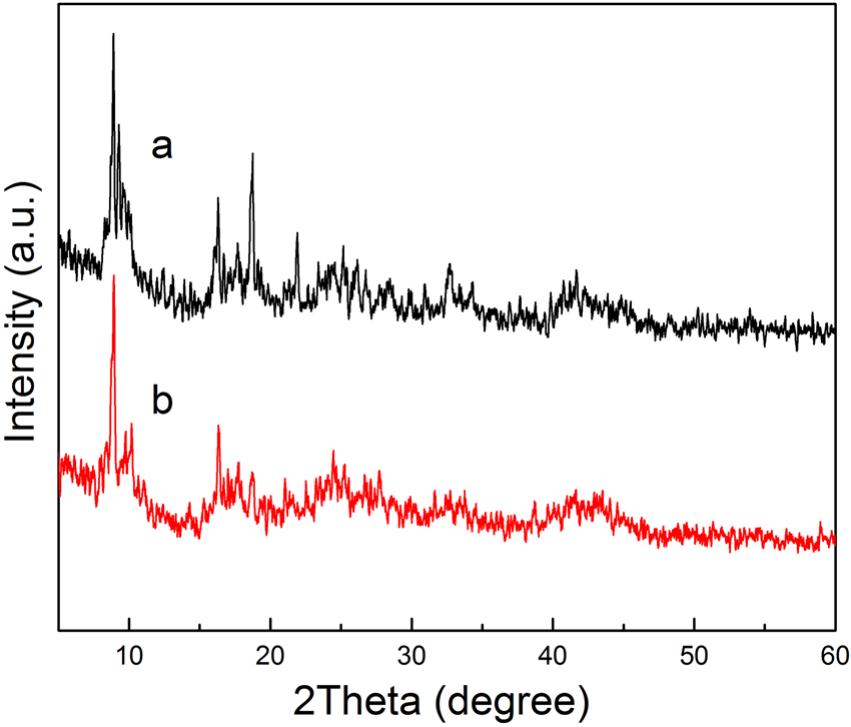
XRD patterns of Fe-based MOFs (**a**) MIL-101(Fe) and (**b**) NH_2_-MIL-101(Fe).

**Figure 2:**
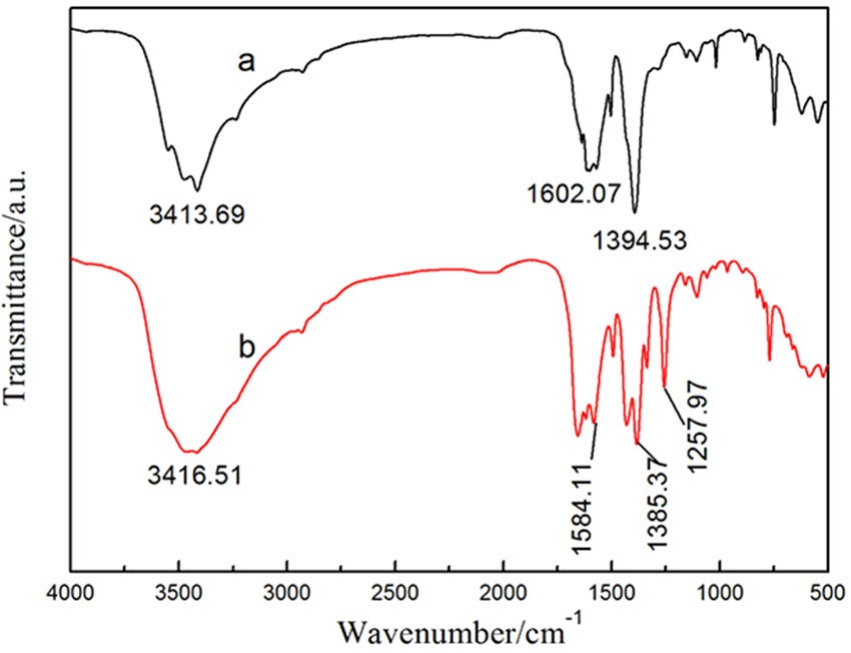
FT-IR spectra of Fe-based MOFs (**a**) MIL-101(Fe) and (**b**) NH_2_-MIL-101(Fe).

**Figure 3:**
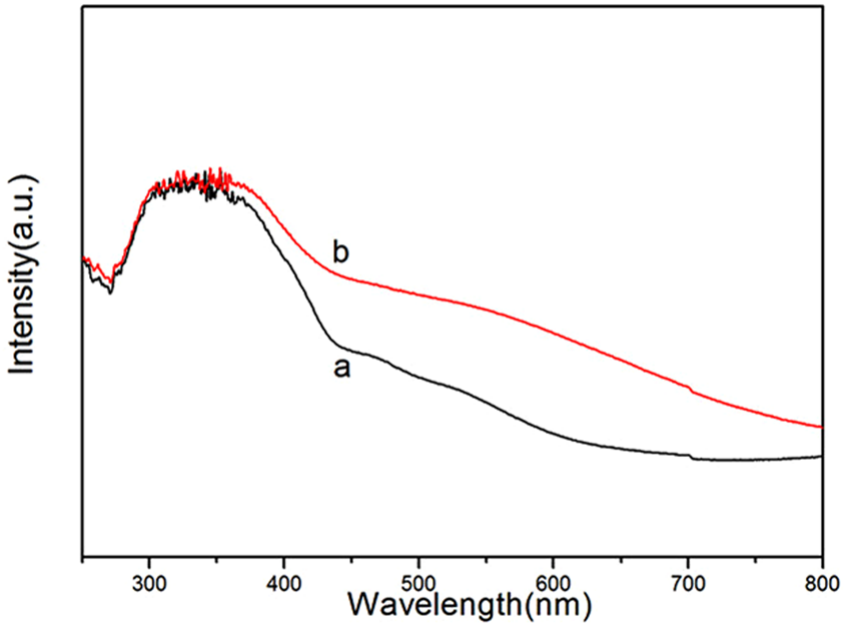
Diffuse reflectance spectra of Fe-based MOFs (**a**) MIL-101(Fe) and (**b**) NH_2_-MIL-101(Fe).

**Figure 4:**
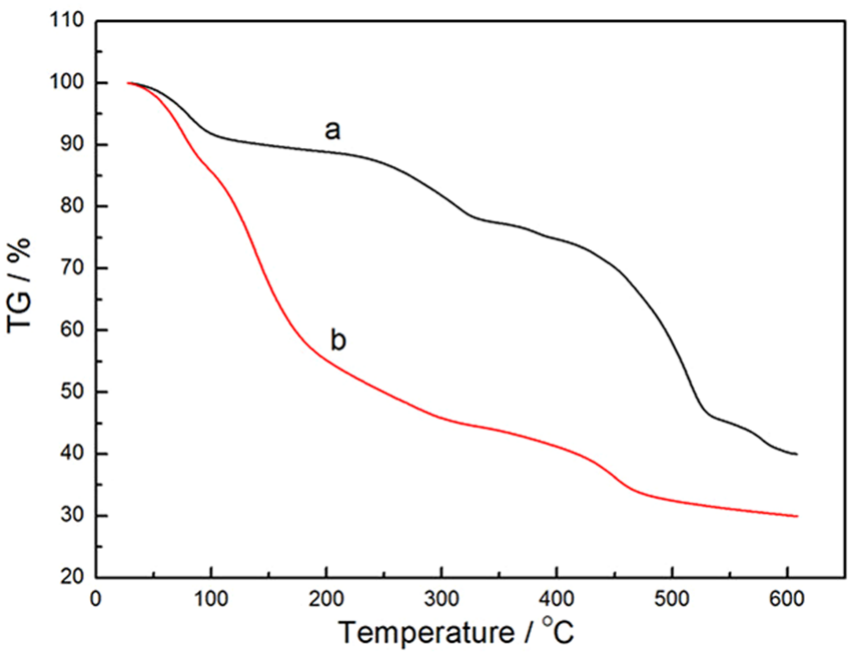
Thermogravimetric curves of Fe-based MOFs (**a**) MIL-101(Fe) and (**b**) NH_2_-MIL-101(Fe).

Figure 1 shows the crystalline structures of MIL-101(Fe) and NH_2_-MIL-101(Fe), and it can be seen that they have extremely similar diffraction patterns. The ligand used in NH_2_-MIL-101(Fe) was NH_2_-H_2_BDC, which is structurally analogous to the H_2_BDC ligand used in MIL-101(Fe). As a result, they have the exact same crystal structure. The XRD peaks correspond to the previously reported diffraction pattern of NH_2_-MIL-101(Fe)^23, 24^, confirming that MIL-101(Fe) and NH_2_-MIL-101(Fe) crystals were well-developed. Figure 2 shows the IR spectra of MIL-101(Fe) and NH_2_-MIL-101(Fe). As illustrated in Fig. 2, these two MIL MOFs consisted of benzene-carboxylates. Thus, the characteristic IR spectra of MIL-101(Fe) and NH_2_-MIL-101(Fe) mainly reflected the benzene-carboxylates. The bands at 1602.07 cm^−1^ and 1584.11 cm^−1^ were attributed to C=O bonding in the carboxylates, and the bands at 1394.53 cm^−1^ and 1385.37 cm^−1^ were from the aromatic carbon C-C vibrational mode, respectively^25^. As the benzene ring in NH_2_-MIL-101(Fe) was substituted with a primary amine, this amine group was observed via the bands at 1257.97 cm^−1^, corresponding to the stretching modes of aromatic carbon C-N bonding^25, 26^. The results of UV-Visible diffuse reflectance spectroscopic analyses of MIL-101(Fe) and NH_2_-MIL-101(Fe) are displayed in Fig. 3. MIL-101(Fe) and NH_2_-MIL-101(Fe) exhibited a clear photo-adsorption edge at the UV light region (~350 nm) because they are photo-catalytically active under UV irradiation^27^. On the other hand, the photo-adsorption edge of NH_2_-MIL-101(Fe) was shifted slightly toward the visible light region. This shift was attributed to the chromophore in NH_2_-H_2_BDC^27^. Thermogravimetric (TG) curves of the MIL MOFs were obtained to further confirm the chemical composition of MOFs, and are displayed in Fig. 4. The TG curves of both MIL-101(Fe) and NH_2_-MIL-101(Fe) exhibited a series of minor weight losses from ambient temperature to 200 °C. The weight loss below 100 °C could be attributed to the removal of water, whereas the weight loss from 100 °C to 250 °C could be due to the elimination of free carboxylic groups^28^. Subsequently, significant weight losses of MIL MOFs were observed from 250 °C to 500 °C owing to the decomposition of ligands^29^. As the temperature exceeded 500 °C, the weight of MIL MOFs remained almost unchanged, suggesting that most of the organic matter had been decomposed by 500 °C. As the NH_2_-MIL-101(Fe) molecule included additional amine groups, it was expected to have a much higher fraction of organic matter. Thus, the residual weight of NH_2_-MIL-101(Fe) was found to be lower than that of MIL-101(Fe).

**Figure 5:**
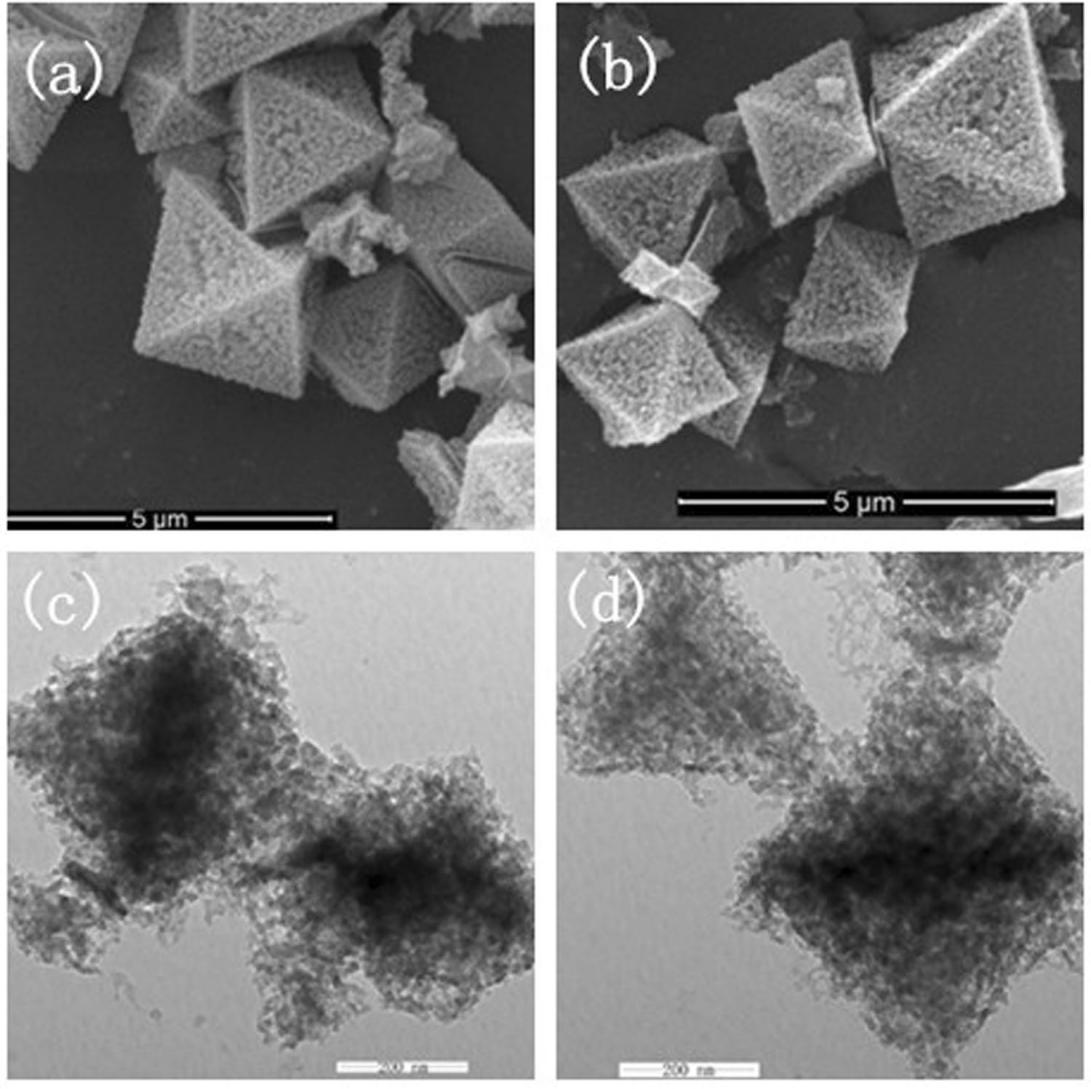
SEM images of (**a**)MIL-101, (b)NH_2_-MIL-101 and TEM images of (**c**)MIL-101(Fe), (**d**) NH_2_-MIL-101(Fe).

**Figure 6:**
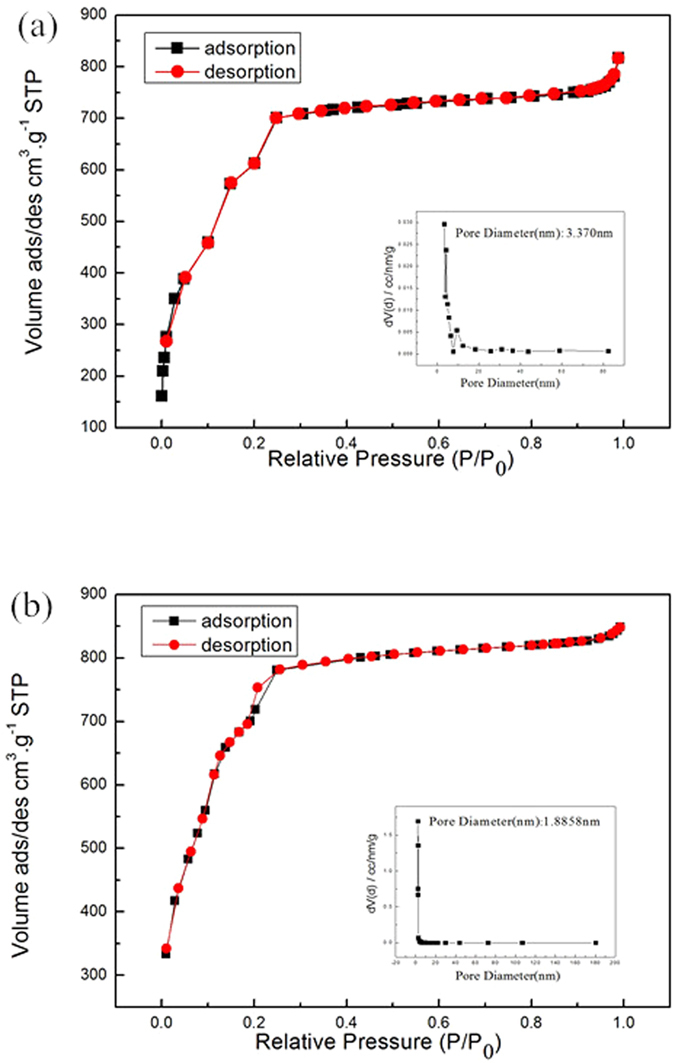
Nitrogen adsorption/desorption isotherm and Barrett-Joyner-Halenda (BJH) pore size distribution plot (inset) of (**a**)MIL-101(Fe) and (**b**)NH_2_-MIL-101(Fe).

The SEM and TEM images of the prepared MIL-101(Fe) and NH_2_-MIL-101(Fe) are shown in Fig. 5. It is obvious that both MIL-101(Fe) and NH_2_-MIL-101(Fe) materials have a loose and uniform polyhedron structure with average diameter of ca. 2–3 μm, as shown in Fig. 5a and b. The unique porous morphologies of the products are characterized by TEM as illustrated in Fig. 5c and d. It can be seen that the as-prepared NH_2_-MIL-101(Fe) retains the original morphology of MIL-101(Fe). The sample surface exhibits a porous frame with hierarchical structure, which is derived from the MOFs. The morphologies of MIL-101(Fe) and NH_2_-MIL-101(Fe) show an octahedral structure, which is similar to the Zr-based materials^20, 21^. The N_2_ adsorption/desorption isotherms and the pore size distributions of the obtained porous MIL-101(Fe) and NH_2_-MIL-101(Fe) products are shown in Fig. 6. The isotherms are identified as type IV, which is characteristic of mesoporous materials. The pore size distribution data indicates that average pore diameters of MIL-101(Fe) and NH_2_-MIL-101(Fe) are 3.37 nm and 1.88 nm, respectively. The BET surface area of the MIL-101(Fe) is 2350.20 m^2^·g^−1^, which increases to as high as 2736.74 m^2^·g^−1^ after the introduction of amine functional groups (Supporting Information, Table S1). Remarkably, the specific surface areas of the samples are comparably higher than most of the previously reported MOFs materials^21, 30, 31^. The large pore sizes and high specific surface areas of the MIL MOFs are expected to contribute greatly to their enhanced adsorption efficiency.

### Adsorption of phosphate on MIL-101(Fe) and NH_2_-MIL-101(Fe)

#### Effects of the adsorbent dosage on the phosphate adsorption

**Figure 7:**
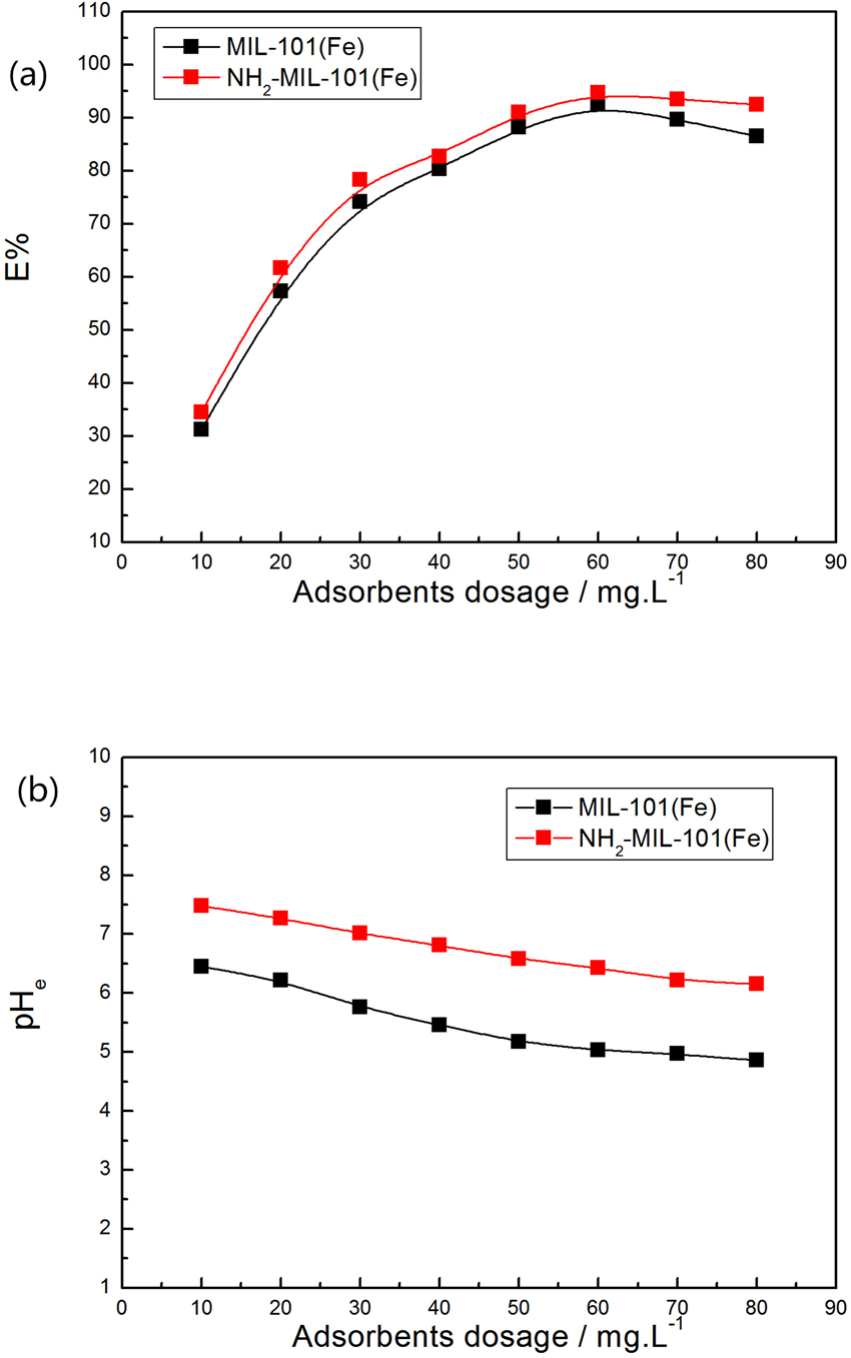
Effects of the adsorbent dosage on (**a**) the removal efficiency E% and (**b**) pH_e_ on Fe-based MOFs (Adsorbents, 2–16 mg; NaH_2_PO_4_ *c*_0_ = 0.6 mg·L^−1^; V = 0.2 L; T = 293 K).

The effect of the adsorbent dosage on phosphate adsorption by the two Fe-based MOFs is illustrated in Fig. 7a. As adsorbent dosages increased from 10 to 60 mg·L^−1^, the phosphate removal efficiency increased for both materials. However, removal efficiency decreased when the adsorbent dosages were higher than 70 mg·L^−1^. Thus, the adsorbent dosage of 60 mg·L^−1^ was chosen for the rest of the adsorption experiments in this study. Phosphate removal efficiency of NH_2_-MIL-101(Fe) was slightly better than that of MIL-101(Fe). At the selected adsorbent dosage (60 mg·L^−1^), phosphate removal efficiencies were 92.50% and 94.67% for the equilibrium total phosphate concentrations of 0.045 mg·L^−1^ and 0.032 mg·L^−1^, respectively, indicating that the Fe-based MOFs have high phosphate adsorption efficiency. Furthermore, the equilibrium pH values (pH_e_) for both materials showed a tendency to drop after the phosphate adsorption (Fig. 7b). The pH_e_ value of MIL-101(Fe) decreased to a weak acid range. For NH_2_-MIL-101(Fe), the pH_e_ value increased slightly above the initial pH (7 ± 0.1), and with the increase in adsorbent dosage, pH_e_ still remained in the neutral range due to the introduction of amine functional groups.

#### Effect of mixing time and kinetics study of the phosphate adsorption

**Figure 8:**
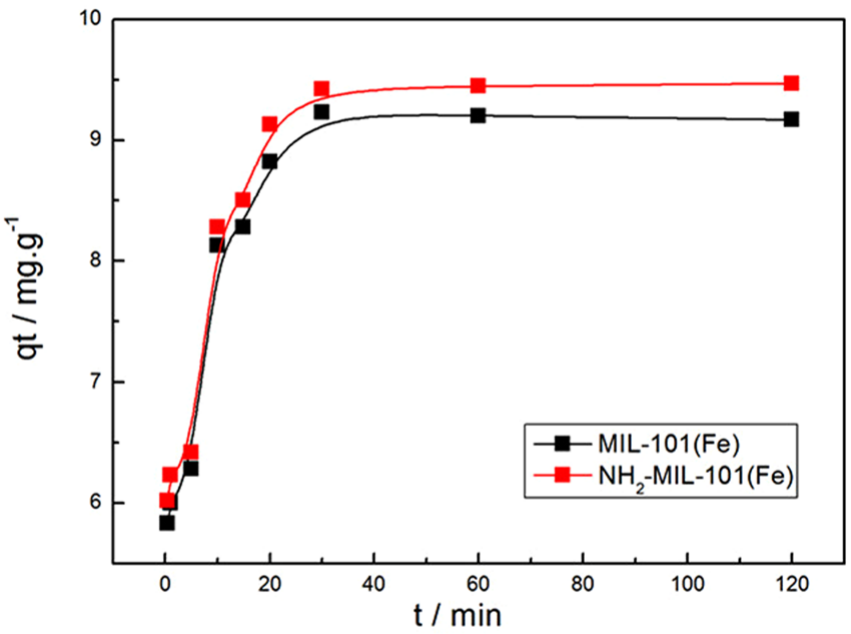
Effects of the mixing time on phosphate adsorption on Fe-based MOFs **(**Adsorbents, 12 mg; NaH_2_PO_4_ *c*_0_ = 0.6 mg·L^−1^; V = 0.2 L; T = 293 K).

**Figure 9:**
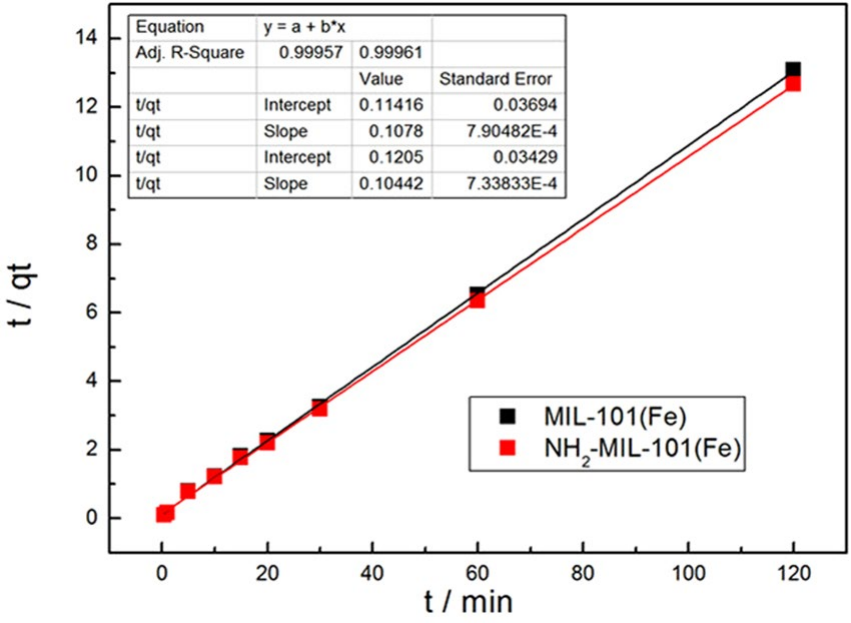
The pseudo second order kinetic equation fitting line of phosphate adsorption on Fe-based MOFs **(**Adsorbents, 12 mg; NaH_2_PO_4_ *c*_0_ = 0.6 mg·L^−1^; V = 0.2 L; T = 293 K).

The phosphate adsorption from aqueous solution on to Fe-based MIL-101 as a function of mixing time is shown in Fig. 8. It can be seen that the adsorption capacity increased rapidly at the start of the adsorption process and then approached a constant value. In the first 30 min, the adsorption capacity of MIL-101(Fe) and NH_2_-MIL-101(Fe) had reached 9.23 mg·g^−1^ and 9.42 mg·g^−1^ respectively, revealing the rapid adsorption of phosphate on Fe-based MIL-101. In order to investigate the adsorption mechanisms and potential rate controlling step of phosphate removal, the pseudo-second-order model was used for describing the process of phosphate adsorption on Fe-based MOFs. The pseudo-second-order equation is generally expressed as Eq. (1):^32^

1 $$\frac{t}{{q}_{t}}=\frac{1}{{k}_{2}{q}_{e}^{2}}+\frac{1}{{q}_{e}}t$$

where *q*_e_ (mg·g^−1^) and *q*_t_ (mg·g^−1^) are the amounts of phosphate adsorbed at equilibrium and at time t (min), respectively; t (min) is the mixing time, and *k*_2_ (g.mmol^−1^·min^−1^) is the pseudo-second-order adsorption rate constant. The fitting of kinetic data using Eq. (1) is shown in Fig. 9. It can be seen that the kinematic data points were well fitted. Correlation coefficients R^2^ were 0.99957 and 0.99961 for the two materials. Moreover, equilibrium adsorption capacity (*q*_e_) calculated from fitting results was consistent with the experimental data of MIL-101(Fe) and NH_2_-MIL-101(Fe) (9.28 and 9.58 mg·g^−1^, respectively). These results suggest that phosphate adsorption on Fe-based MIL-101 follows the pseudo-second-order model, which was developed based on the assumption that the rate limiting step may be a chemisorption process involving valency forces via sharing (or exchange) of electrons between adsorbate and adsorbent^33, 34^. Model constants for adsorption on MIL-101(Fe) and NH_2_-MIL-101(Fe) derived from the pseudo-second-order equation are also listed in Table S2 (Supporting Information). While the *k*_2_ of MIL-101(Fe) was found to be higher than the *k*_2_ of NH_2_-MIL-101(Fe), the estimated *q*_e_ (i.e., *q*_e,est_) from the fitting of NH_2_-MIL-101(Fe) was relatively high compared to that of MIL-101(Fe). The difference was also observed from the experimental data as shown in Fig. 8. This might be due to the presence of NH_2_ in NH_2_-MIL-101(Fe), which could attract phosphate ions through electrostatic interactions.

#### Adsorption isotherms of phosphate adsorption on Fe-based MOFs

**Figure 10:**
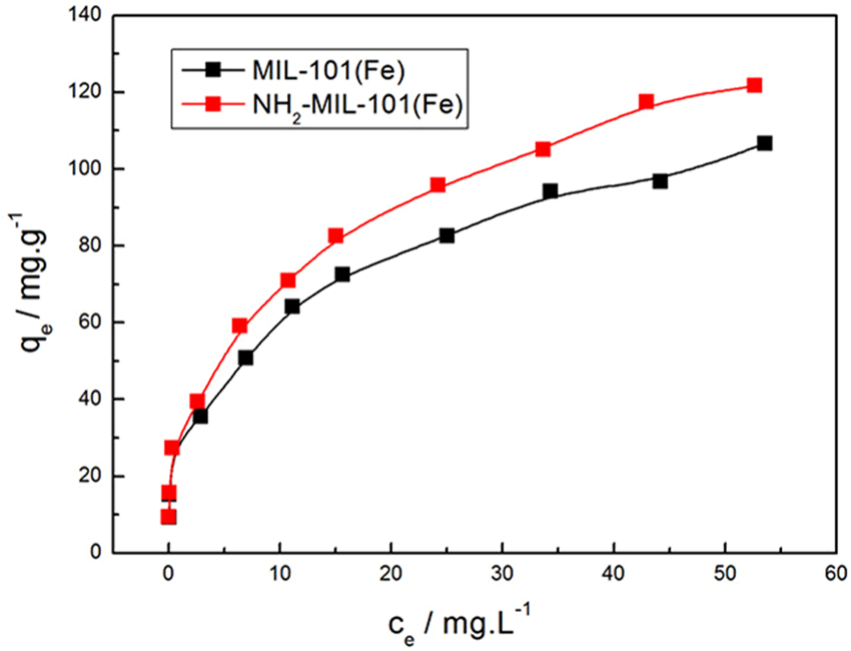
Adsorption isotherms of phosphate on Fe-based MIL-MOFs **(**Adsorbents, 12 mg; NaH_2_PO_4_ *c*_0_ = 0.6–60 mg·L^−1^; V = 0.2 L; T = 293 K).

The adsorption isotherms of phosphate on the Fe-based MOFs are shown in Fig. 10. The *q*_e_ increased correspondingly with the increase in *c*_e_ and approached a constant value. Adsorption isotherm models were employed for describing the equilibrium studies. The Langmuir and Freundlich models were used for fitting the equilibrium data. The Langmuir model assumes that the adsorption occurs as a mono-layer on a homogenous surface, where the number of adsorptive sites is finite. Once the adsorptive sites are occupied, they cannot adsorb other adsorbates. Therefore, maximal adsorption capacity (*q*_max_) is expected. The Langmuir model is expressed as Eq. (2):^35, 36^

2 $$\frac{{c}_{e}}{{q}_{e}}=\frac{1}{{q}_{m}{K}_{L}}+\frac{1}{{q}_{m}}{c}_{e}$$

where *K*_L_ (L·mg^−1^) represents the Langmuir adsorption constant, associated with the adsorption bonding energy. Furthermore, the separation constant *R*_L_ is another important property of the Langmuir adsorption isotherm which is used to characterize the degree to which the adsorption reaction proceeded, and the expression is shown as Eq. (3):^37^

3 $${R}_{L}=\frac{1}{(1+{K}_{L}{C}_{0})}$$

The Freundlich model is applicable for the heterogeneous surface of adsorbent with multilayer adsorption. In this model, the maximum adsorption capacity is uncertain and adsorption sites are not equal with different energies. The Freundlich model is expressed as Eq. (4):^38^

4 $$\mathrm{ln}\,{q}_{e}=\,\mathrm{ln}\,{K}_{f}+\frac{1}{n}\,\mathrm{ln}\,{c}_{e}$$

where *K*_*f*_ (mg·g^−1^(L·mg^−1^)^1/n^) is the Freundlich constant related to the adsorption capacity of the adsorbent, and 1/*n* signifies adsorption intensity. The value of 1/*n* indicates whether the type of isotherm is favorable (1/*n* < 1) or unfavorable (1/*n* > 2)^39^.

**Figure 11:**
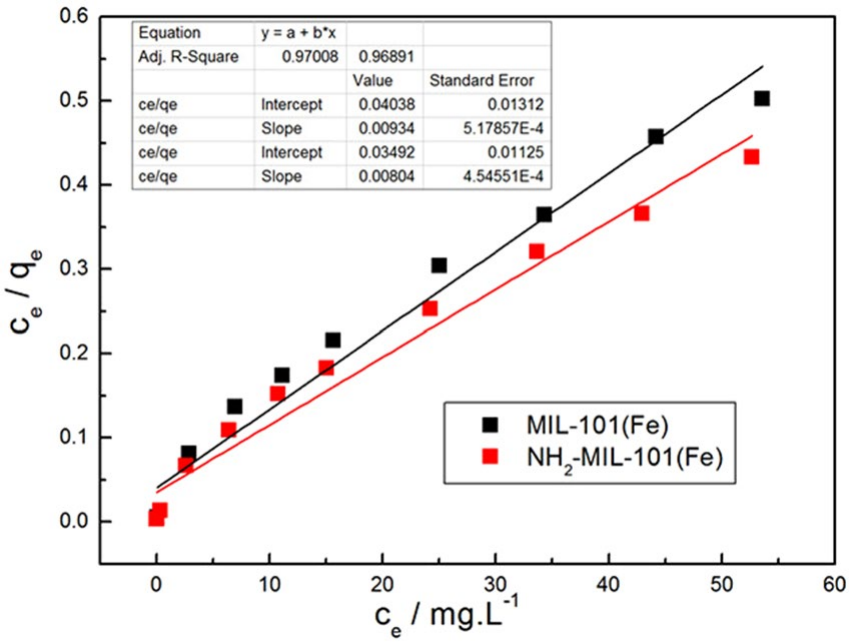
Langmuir plots of the isotherms for phosphate adsorption on Fe-based MIL-MOFs **(**Adsorbents, 12 mg; NaH_2_PO_4_ *c*_0_ = 0.6–60 mg·L^−1^; V = 0.2 L; T = 293 K).

**Figure 12:**
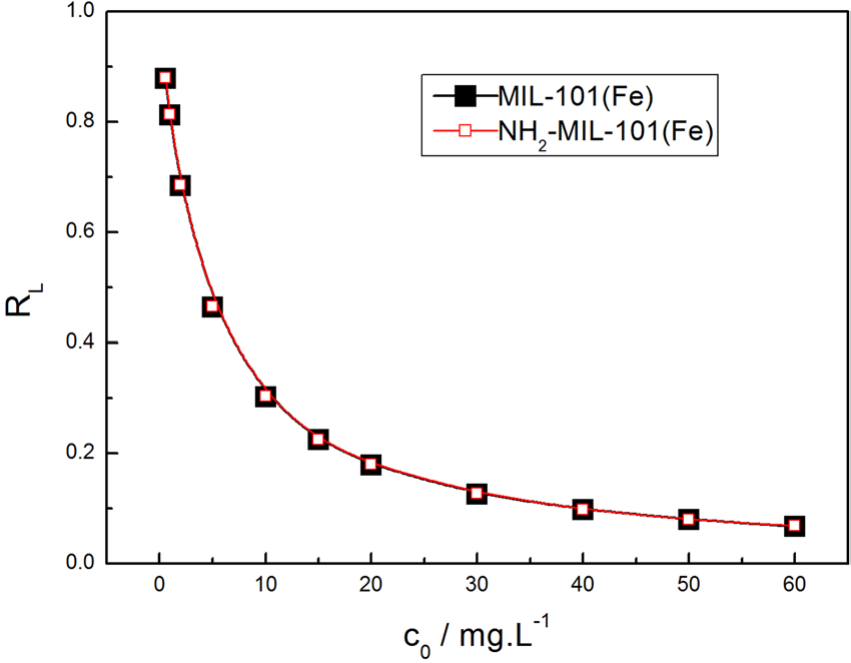
Plot of separation factor (*R*_L_) versus initial phosphate concentration(*c*_0_) **(**Adsorbents, 12 mg; NaH_2_PO_4_ *c*_0_ = 0.6–60 mg·L^−1^; V = 0.2 L; T = 293 K).

**Figure 13:**
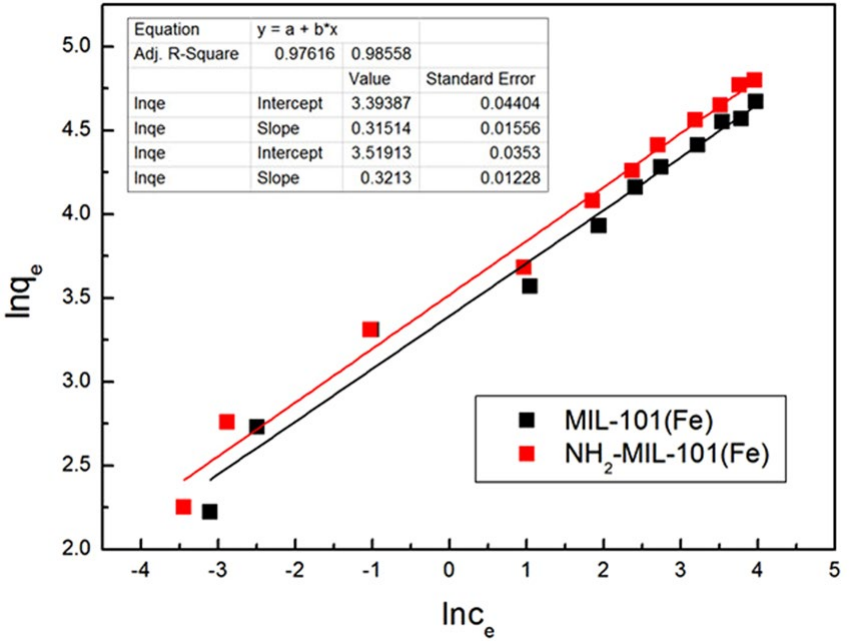
Freundlich plots of the isotherms for phosphate adsorption on Fe-based MOFs **(**Adsorbents, 12 mg; NaH_2_PO_4_ *c*_0_ = 0.6–60 mg·L^−1^; V = 0.2 L; T = 293 K).

As shown in Fig. 11, the Langmuir isotherm exhibited a better fit with higher correlation coefficients for MIL-101(Fe) and NH_2_-MIL-101(Fe) (R^2^ = 0.97008 and 0.96891, respectively). Furthermore, the *q*_m_ calculated by this function was quite close to the values actually determined. The maximum adsorption capacities of MIL-101(Fe) and NH_2_-MIL-101 are calculated to be 107.70 mg·g^−1^ and 124.38 mg·g^−1^, respectively. These values are consistent with the results observed in the adsorption kinetics experiment. NH_2_ was found to be a suitable functional group to enhance the phosphate adsorption. In addition, it can be seen from Fig. 12 that *R*_L_ decreased with the increase in initial concentration of phosphate, demonstrating that increase in the adsorbate concentration is favorable for the adsorption. Figure 13 represents the fitting of the isotherm data of Fe-based MIL-101 using the Freundlich model. Correlation coefficients for adsorption on MIL-101(Fe) and NH_2_- MIL-101(Fe) were 0.97616 and 0.98558, respectively. The related parameters and correlation coefficients (R^2^) of the two models are tabulated in Table S2 (Supporting Information). Moreover, from the R^2^ values and fitting lines, we also found that the *n* values were larger than 1, indicating that the adsorption process readily occurred on MIL-101(Fe) and NH_2_- MIL-101^39^.

#### Effect of co-existing ions and adsorption of phosphate from real water samples

**Figure 14:**
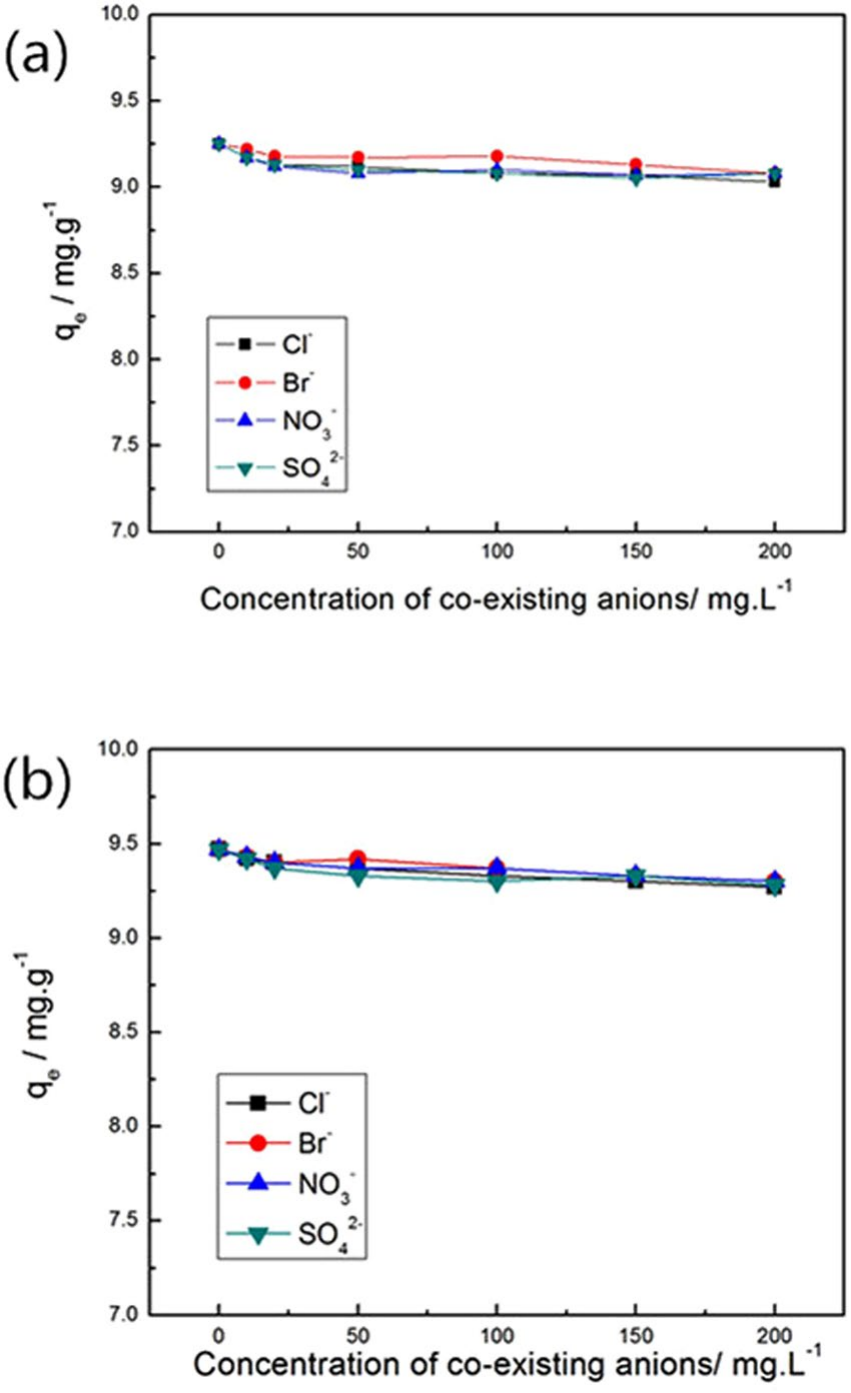
Effect of co-existing anions on the phosphate adsorption on (**a**) MIL-101(Fe) and (**b**) NH_2_-MIL-101(Fe) **(**Adsorbents, 12 mg; NaH_2_PO_4_ *c*_0_ = 0.6 mg·L^−1^; V = 0.2 L; T = 293 K).

Figure 14a and b show the phosphate adsorption on MIL-101(Fe) and NH_2_-MIL-101(Fe), respectively, in the presence of other anions including chloride, bromide, nitrate and sulfate ions. Figure 14 reveals that when the other anions were present in the phosphate solution with concentration of each ion species between 10 to 200 mg·L^−1^, the *q*_e_ values of MIL-101(Fe) and NH_2_-MIL-101(Fe) showed a slight decreasing trend. However, the removal efficiency of phosphate still remained above 90%, indicating that the presence of chloride, bromide, nitrate and sulfate ions did not hinder the adsorption of phosphate on Fe-based MIL-101. Furthermore, the adsorption of chloride, bromide, nitrate and sulfate ions on Fe-based MIL-101 was negligible, showing that the Fe-based MIL-101 materials possessed a high selectivity towards phosphate over the other anions.

**Figure 15:**
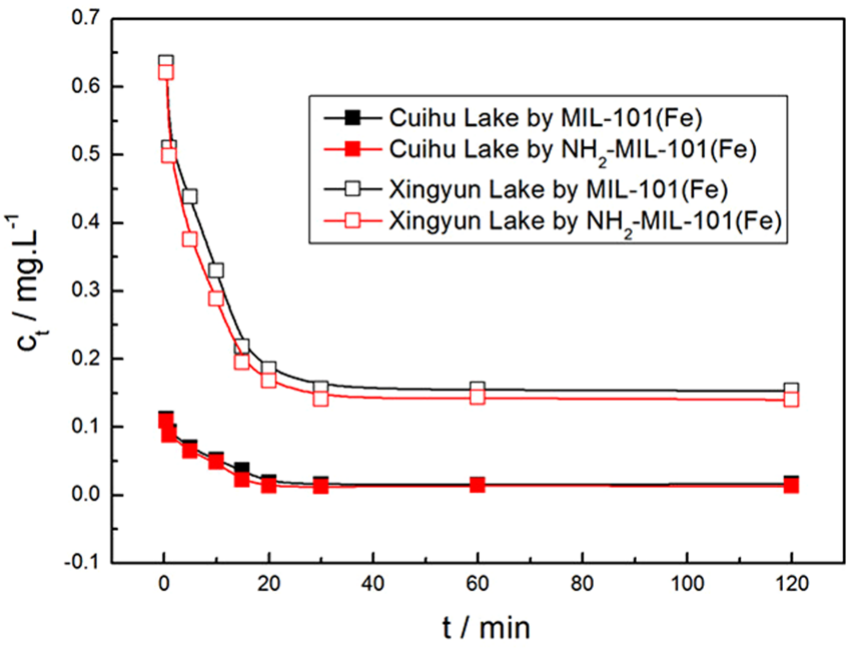
Adsorption of phosphate on MIL-101(Fe) and NH_2_- MIL-101(Fe) in two real eutrophic water samples (Cuihu Lake, *c*_0_ = 0.265 mg·L^−1^; Xingyun Lake, *c*_0_ = 1.561 mg·L^−1^; Adsorbents, 12 mg; V = 0.2 L; T = 293 K).

The removal of phosphate from water can significantly reduce eutrophication issues in lakes and reservoirs. Thus, in this study, we examined the feasibility of using the Fe-based MIL-101 materials to remove phosphate from two real eutrophic water samples (Cuihu Lake and Xingyun Lake in Yunnan Province, China). Figure 15 shows the remaining concentrations of phosphate in the eutrophic water samples after a certain mixing time. When the initial phosphate concentration c_0_ was 0.265 mg·L^−1^, both MIL-101 and NH_2_-MIL-101 were able to completely remove phosphate from the eutrophic water samples within 20 min. This reveals that the Fe-based MIL-101 adsorbents are capable of removing the low-concentration phosphate from eutrophic water samples effectively and rapidly. When c_0_ was 1.561 mg·L^−1^, phosphate was partially removed by Fe-based MIL-101 and the kinetics were relatively slow. The corresponding kinetic constants and estimated *q*_e_ for the phosphate adsorption from eutrophic water samples by Fe-based MIL-101 are summarized in Table 1. The kinetic constants confirm that the adsorption kinetics were much slower for the phosphate adsorption with c_0_ = 1.561 mg·L^−1^ than the adsorption with c_0_ = 0.265 mg·L^−1^.

**Table 1:** Model constants for phosphate adsorption on MIL-101 and NH_2_-MIL-101 from real eutrophic water samples, derived from the pseudo-second-order equation.

Adsorbents	*c*_0_/mg·L^−1^	*k*_2_/g·mg^−1^·min^−1^	*q*_e_/mg·g^−1^	R^2^
MIL-101(Fe)	0.265	0.309	4.18	0.99967
	1.561	0.059	23.62	0.99985
NH_2_-MIL-101(Fe)	0.265	0.349	4.23	0.99978
	1.561	0.069	23.82	0.99991

#### Recyclability and Desorption of Fe-based MOFs for Phosphate Adsorption

**Figure 16:**
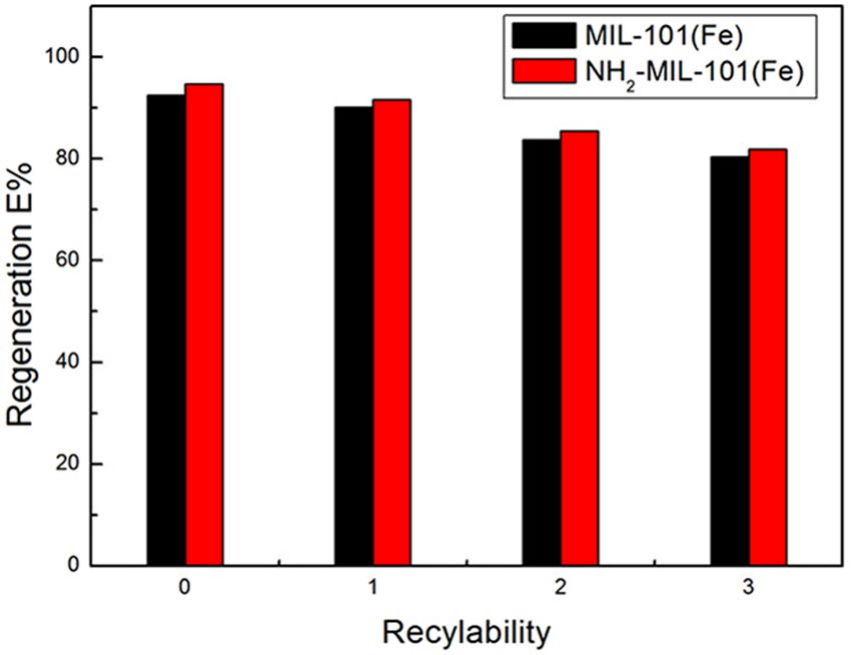
Recyclability tests in phosphate adsorption on Fe-based MOFs.

Recyclability of Fe-based MOFs is also a critical property demanding investigation. To regenerate Fe-based MOFs, the spent adsorbents were soaked in a concentrated sodium chloride solution (1%) and stirred at ambient temperature for 24 h. The washed Fe-based MIL-101 materials were dried and then used for the phosphate adsorption. Figure 16 displays the regeneration efficiency of Fe-based MIL-101 after a few cycles of regeneration. Despite the slight drop in the regeneration efficiency after the first cycle, Fe-based MOFs still exhibited considerable adsorption efficiency (above 85%) for the subsequent cycles. This reveals that Fe-based MOFs can be regenerated and reused multiple times.

Table 2 shows that the desorption of adsorbed phosphate (D) by Fe-based MOFs increased with increasing amount of phosphate adsorption (A). From Table 2, we can see clearly that the desorption rate (D/A) of adsorbed phosphate is very low. When the initial phosphate concentration was 60 mg·L^−1^, the phosphate desorption rate of MIL-101(Fe) was only 1.97%, while the phosphate desorption rate of NH_2_-MIL-101(Fe) was only 1.78%. The maximum desorption rate was less than 2%, which showed that more than 98% of the phosphate was adsorbed through specific adsorption, and it was difficult to desorb.

**Table 2:** The ratio of desorbed P to adsorbed P for Fe-based MIL MOFs.

c_0_/mg·L^−1^	MIL-101(Fe)			NH_2_-MIL-101(Fe)		
	A/mg·g^−1^	D/mg·g^−1^	D/A%	A/mg·g^−1^	D/mg·g^−1^	D/A %
0.6	9.25	0	0	9.47	0	0
1.0	15.28	0.10	0.65	15.73	0.13	0.83
2.0	27.28	0.22	0.81	27.33	0.25	0.91
5.0	35.50	0.50	1.41	39.50	0.55	1.39
10.0	50.83	0.77	1.51	59.17	0.87	1.47
15.0	64.17	0.97	1.51	70.83	1.08	1.52
20.0	72.50	1.20	1.38	82.50	1.20	1.45
30.0	82.50	1.27	1.54	95.83	1.30	1.36
40.0	94.17	1.52	1.61	105.00	1.53	1.46
50.0	96.67	1.70	1.76	117.5	1.82	1.55
60.0	106.67	1.97	1.85	121.67	2.17	1.78

A-Adsorbed phosphate(mg·g^−1^); D-Desorbed phosphate(mg·g^−1^); D/A-Desorption rate(%).

#### Proposed mechanism of phosphate adsorption on Fe-based MOFs

**Figure 17:**
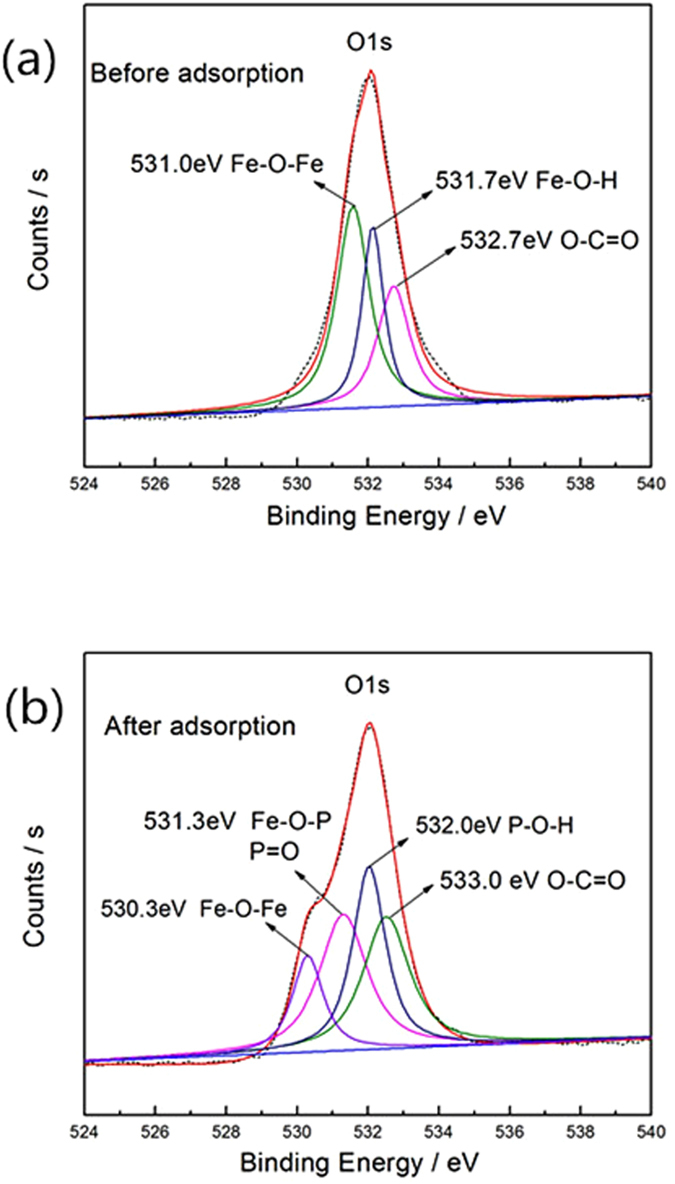
O1s XPS spectra of the MIL-101(Fe) (**a**) before and (**b**) after phosphate adsorption.

**Figure 18:**
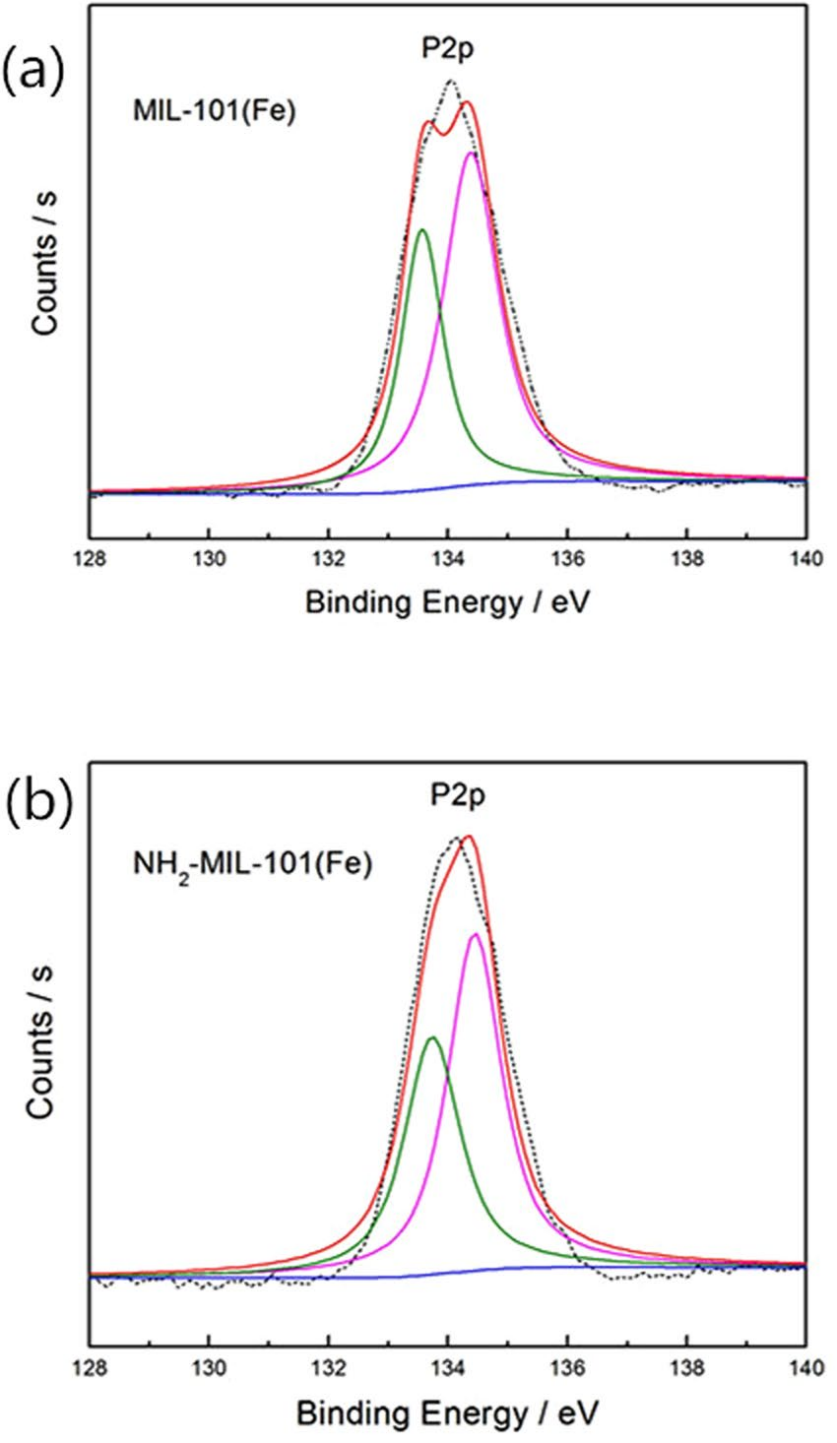
P2p XPS spectra of the (**a**) MIL-101(Fe) and (**b**) NH_2_-MIL-101(Fe) adsorption of phosphate.

The analyses of the adsorption isotherms indicate that phosphate adsorption on both MIL-101(Fe) and NH_2_-MIL-101(Fe) involved chemical interactions. It was observed that the presence of the amine group significantly enhanced phosphate adsorption on NH_2_-MIL-101(Fe), possibly via electrostatic attraction. However, the increased adsorption only accounted for approximately 10% of the entire adsorption capacity. In addition, MIL-101(Fe), which contained no amine groups, still exhibited considerable adsorption capacity. Thus, there must be other chemical affinities between phosphate and MIL-101. It has been reported that the metal sites in MOFs can be partially positively-charged^20, 21, 40, 41^. These metal sites could interact with negatively-charged sites via the electrostatic interaction. As Fe in MIL-101(Fe) and NH_2_-MIL-101(Fe) was in the form of iron oxide, the interaction between Fe and phosphate may affect the local order of the Fe-O bonding. Thus, we also measured the XPS spectra of O, Fe and P adsorbed on Fe-based MOFs to further investigate the interaction between Fe-based MOFs and adsorbates. For the MIL-101 after phosphate adsorption, the O1s spectrum (Fig. 17b) consists of four peaks, which could be assigned to O in O-C=O (533.0 eV), P-O-H (532.0 eV), Fe-O-Fe (530.3 eV) in Fe−O−P and P=O (531.3 eV), respectively^42^. Similar peaks can be observed in the deconvoluted O1s spectrum of NH_2_-MIL-101 (Supporting information Fig. S1b). These data strongly verify that the Fe-OH groups in Fe-based MIL-101 present high affinity toward phosphate molecules, and also suggest that Fe was successfully bonded to oxygen atoms of H_2_BDC or NH_2_-H_2_BDC. Figure 18a and b show the P2p XPS spectra of the MIL-101(Fe) and NH_2_-MIL-101(Fe) adsorption of phosphate. The P2p peak can be split into two peaks of P2p1/2 and P2p3/2 with binding energies of 134 eV and 133 eV^43^, respectively, confirming that phosphate has been successfully adsorbed on the Fe-based MIL-101 material. This result also suggests that chemisorption is likely involved in the phosphate adsorption process, in line with the kinetic analysis. Considering these interactions, a possible mechanism for the phosphate adsorption on Fe-based MIL-101 was proposed, which suggests that phosphate can be attracted to MIL-101(Fe) via the interaction between phosphate ions and Fe metal. NH_2_-MIL-101(Fe) in particular could provide another adsorptive site to attract phosphate via the amine and phosphate interaction.

## Conclusions

Two Fe-based MOFs, MIL-101(Fe) and NH_2_-MIL-101(Fe), were synthesized and used for phosphate adsorption in water and real eutrophic water samples. NH_2_-MIL-101(Fe) was found to exhibit higher adsorption capacities than MIL-101(Fe), possibly due to the amine group in NH_2_-MIL-101(Fe) which attracted phosphate ions. However, interaction between phosphate and Fe sites of Fe-based MOFs might be the primary factor accounting for the phosphate adsorption on Fe-based MOFs. The Fe-based MOFs also exhibited a high selectivity towards phosphate over other anions such as chloride, bromide, nitrate and sulfate. Furthermore, the Fe-based MOF materials were also capable of adsorbing phosphate in actual eutrophic water samples and showed better removal effect. We also found that Fe-based MIL-101 could be easily regenerated and reused for phosphate adsorption. The adsorption of phosphorus was specific adsorption, and not easy to desorb. These findings reveal that Fe-based MOFs can be effective and selective adsorbents for the removal of phosphate from eutrophic water bodies.

## Experimental Section

### Materials and Methods

#### Synthesis of MIL-101(Fe) and NH_2_- MIL-101(Fe)

The MIL-101(Fe) was prepared using the procedure described by Nataliya^44^. Briefly, 0.675 g (2.45 mmol) of FeCl_3_·6H_2_O and 0.206 g (1.24 mmol) of terephthalic acid (H_2_BDC) were dissolved in 30 mL of DMF. After thermal treatment in a Teflon-lined autoclave for 20 h at 110 °C, the reaction product was recovered by centrifugation and washed several times with DMF and ethanol. Then the obtained solid was finally dried for 8 h at 150 °C. The NH_2_-MIL-101(Fe) material was prepared according to the same protocol, except that 0.225 g (1.24 mmol) of 2-aminoterephthalic acid (NH_2_-H_2_BDC) was used instead of H_2_BDC^45^.

#### Characterization

X-ray diffraction (XRD) was carried out to identify the phase composition of synthesized samples over the 2θ range from 3° to 90° using a Rigaku D/max-A diffractometer with Co Kα radiation. A Fourier transform infrared spectrometer (FTIR, Thermo Nicolet 670FT-IR) was used for recording the FTIR spectra of the samples over the range 400 to 4000 cm^−1^. UV–Visible diffuse reflectance spectra were measured on UV–Visible spectrophotometer (Shimadzu Z-2000) with BaSO_4_ as the reflectance standard reference. Thermogravimetric analysis (TGA) was conducted on a STA449 F3 TG-DSC thermal analyzer (Netzsch, Germany) heating the sample to 800 °C under air atmosphere at a heating rate of 5 °C/min. Morphologies of the synthesized samples were observed with an AMRAY 1000B scanning electron microscope (SEM), and the microstructural characteristics of samples were observed by high-resolution transmission electron microscope (HR-TEM, JEOL JEM-2010) working at 200 kV accelerating voltage. The lattice structure was identified by selected area electron diffraction (SAED) technique. Nitrogen adsorption-desorption measurements were conducted at 77 K on a Micromeritics Tristar apparatus. Specific surface areas were determined following the Brunauer-Emmett-Teller (BET) analysis. X-ray photoelectron spectroscopy (XPS) was performed on a PH1500 Versaprobe-II spectrometer using monochromatized Al Kα at hυ = 1486.6 eV. A precision pH meter (Mettler-Toledo) was used to measure the pH values of solutions.

#### Adsorption of phosphate on MIL-101(Fe) and NH_2_-MIL-101(Fe)

Adsorption behaviors of phosphate on Fe-based MOFs were studied using batch-type adsorption experiments. A certain amount of Fe-based MOFs was added to 0.2 L of phosphate solution with a given initial concentration (*c*_0_). The resulting mixture was subsequently placed in a temperature-controllable magnetic stirrer at 200 rpm to begin the adsorption. The adsorption experiments were performed for 30 min to ensure that the adsorption reached the equilibrium. The adsorption capacity at equilibrium was denoted as *q*e (mg·g^−1^) and calculated using the following equation (Eq. (5)):

5 $${q}_{{\rm{e}}}=\frac{({c}_{0}-{c}_{e})V}{m}$$

The phosphate removal efficiency (E%) of Fe-based MOFs was calculated using the following equation (Eq. (6)):

6 $$E \% =\frac{{c}_{0}-{c}_{e}}{{c}_{0}}\times 100 \% $$

Where m (g) is the weight of Fe-based MOFs, *c*_e_ (mg·L^−1^) is equilibrium concentration of phosphate and *V* represents the total volume of solution. The concentration of phosphate in supernatant was analyzed by the National Standard Method of China (GB11893-89): Water quality-Determination of total phosphate-Ammonium molybdate spectrophotometric method.

#### Effect of co-existing ions and adsorption of phosphate from real water sample

To examine the effect of co-existing anions on the adsorption of phosphate on Fe-based MOFs, solutions containing different concentrations of chloride, bromide, nitrate and sulfate ions were prepared with the concentration ranging from 10 to 200 mg·L^−1^.

Removal of phosphate from real water samples was also examined. Water samples from Cuihu Lake and XingYun Lake were used in this investigation. Cuihu Lake^46^ is an urban landscape lake in Kunming, Yunnan, China. The surface area of the lake is approximately 15 × 10^4^ m^2^, with a mean depth of 1.5 m. Total P (TP) concentration is 0.265 mg·L^−1^ and the average pH value is 8.0. Since the 1990s, the lake water quality has gradually become eutrophic because of urban sewage inflow, tourist behavior and habitation of black-headed gull. XingYun Lake^47, 48^ is located in north Jiangchun County, Yunnan Province, China. It is one of the nine largest plateau freshwater lakes in Yunnan. The lake is approximately 34.33 km^2^ in surface area, with a mean depth of 7 m, total P (TP) concentration of 1.561 mg·L^−1^ and pH value averaging 9.5 at the time of sampling. Xingyun lake has a much higher eutrophication degree than Cuihu Lake. According to the Chinese Surface Water Environmental Quality Standard^46^, water quality of Cuihu Lake belongs to class III, while XingYun Lake has inferior class V water quality.

#### Desorption of phosphate from loaded Fe-based MIL MOFs

After the adsorption test, the Fe-based MOF material was washed with anhydrous ethanol, and centrifuged. The supernatant was discarded. The phosphate loaded adsorbent was added to 0.2 L of 0.01 mol·L^−1^ CaCl_2_ solution, and subsequently the mixture was oscillated for 1 hour at ambient temperature, and then desorbed for 23 h. The suspension was centrifuged for 10 min at 5000 rpm, and the total phosphate concentration in the supernatant was measured.

## Electronic supplementary material

Supporting information
