# Supplementary material for: Effective Adsorption and Removal of Phosphate from Aqueous Solutions and Eutrophic Water by Fe-based MOFs of MIL-101
Source: Sci Rep. 2017 Jun 12;7:3316. doi: 10.1038/s41598-017-03526-x (PMC5468308; doi:10.1038/s41598-017-03526-x)
Supplement: Supplementary file 1 — Supporting information [file 41598_2017_3526_MOESM1_ESM.pdf]

Supporting information

Effective Adsorption and Removal of Phosphate from Aqueous Solutions and Eutrophic Water by Fe-based MOFs of MIL-101

Qiying Xie, Yan Li, Zhaoling Lv, Hang Zhou, Xiangjun Yang, Jing Chen, Hong Guo*

School of Chemistry Science and Engineering, Yunnan University, Kunming 650091, Yunnan, China

Yunnan Key Laboratory of Micro/Nano Materials & Technology, Kunming 650091, Yunnan, China

Electronic Supplementary Information

Table S1 Surface areas and pore volumes of MIL-101(Fe) and NH_2-MIL-101(Fe)

	MIL-101(Fe)	NH_2-MIL-101
Surface areas(m^2 g^{-1})	2350.20	2736.74
pore volumes(cm^3 g^{-1})	0.131	0.746

Table S2 Model constants for phosphate adsorption on MIL-101 and NH_2-MIL-101 derived from the pseudo-second-order equation

Adsorbents	k_2 / g ·mg^{-1} ·min^{-1}	q_e /mg g^{-1} (fitted value)	q_e /mg g^{-1} (experimental value)	R^2
MIL-101(Fe)	0.102	9.28	9.20	0.99957
NH_2-MIL-101(Fe)	0.090	9.58	9.45	0.99961

Table S3 Adsorption isotherm model constants derived from Langmuir and Freundlich isotherms

Adsorbents	Langmuir			Freundlich		
	K_L / L·mg^{-1}	q_m/ mg g^{-1}	R^2	K_f / mg g^{-1}(L·mg^{-1})$^{1/n}$	n	R^2
MIL-101(Fe)	0.231	107.07	0.97008	29.78	3.17	0.97616
NH_2-MIL-101(Fe)	0.230	124.38	0.96891	33.76	3.11	0.98558

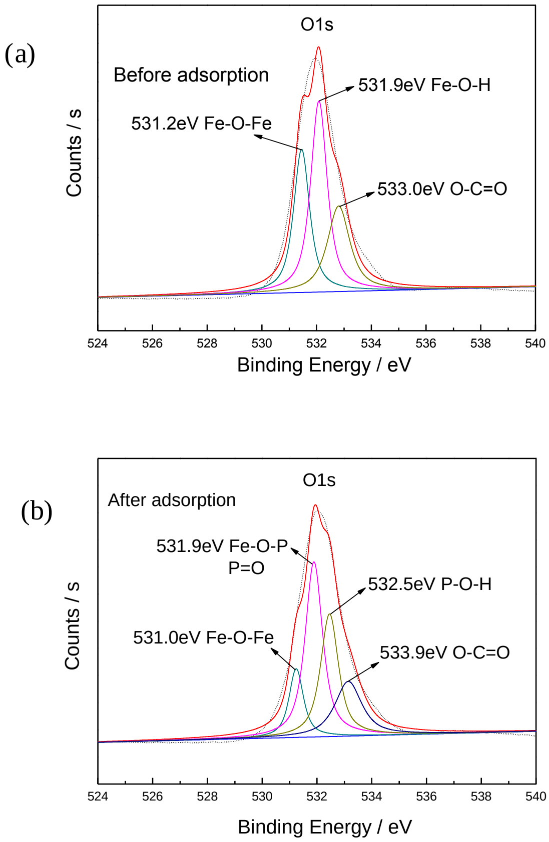

Figure S1. O1s XPS spectra of the NH_2-MIL-101(Fe) (a) before and (b) after phosphate adsorption
